# Voltammetric Behaviour of Rhodamine B at a Screen-Printed Carbon Electrode and Its Trace Determination in Environmental Water Samples

**DOI:** 10.3390/s22124631

**Published:** 2022-06-19

**Authors:** Kevin C. Honeychurch

**Affiliations:** Faculty of Applied Sciences, University of the West of England, Bristol, Frenchay Campus, Coldharbour Lane, Bristol BS16 1QY, UK; kevin.honeychurch@uwe.ac.uk; Tel.: +44-1173287357

**Keywords:** Rhodamine B, screen-printed carbon electrode, voltammetry, water

## Abstract

The voltammetric behaviour of Rhodamine B was studied at a screen-printed carbon electrode (SPCE), by cyclic and differential pulse voltammetry. Cyclic voltammograms exhibited two reduction peaks (designated R1 and R2) generated from the reduction of the parent compound through, first, one electron reduction (R1) to give a radical species, and then a further one-electron, one-proton reduction to give a neutral molecule (R2). On the reverse positive-going scan, two oxidation peaks were observed. The first, O1, resulted from the oxidation of the species generated at R2, and the second, O2, through the one-electron oxidation of the amine group. The nature of the redox reactions was further investigated by observing the effect of scan rate and pH on the voltammetric behaviour. The developed SPCE method was evaluated by carrying out Rhodamine B determinations on a spiked and unspiked environmental water sample. A mean recovery of 94.3% with an associated coefficient of variation of 2.9% was obtained. The performance characteristics indicated that reliable data may be obtained for Rhodamine B measurements in environmental water samples using this approach.

## 1. Introduction

**Scheme 1:**
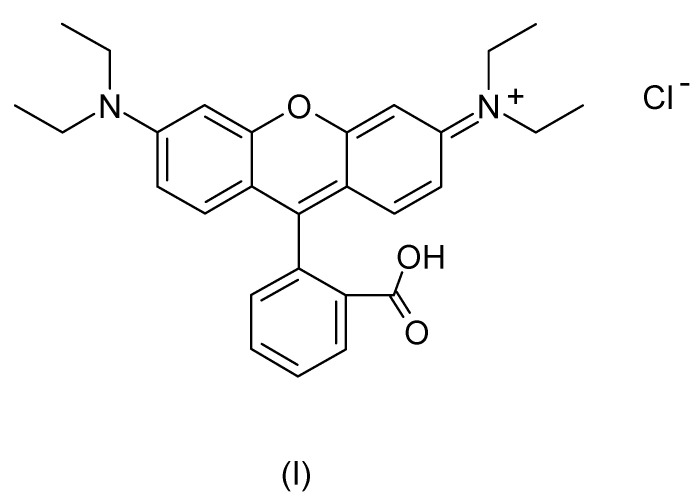
Structure of Rhodamine B.

Rhodamine B (I) Scheme 1 is widely used in industry as a dye for plastics, wool, silk and paper [1]. Numerous reports have also highlighted its use as a florescent dye in many diverse scientific applications (i.e., [2,3,4,5,6]). However, in more recent years the mutagenic activity of this compound has become more evident [7]. Rhodamine B has been reported to exhibit mutagenic activity and DNA damage in certain cell lines [8]. It has also been found to be toxic to oyster larvae and eggs at levels greater than 1 µgmL^−1^ [9] with higher levels shown to be toxic to algae [10]. Studies on the effects on rats have shown growth retardation, decreased reproductive ability, behavioural effects, increased susceptibility to infection and tumour development [11]. As a result, its use as a dye in food has been prohibited in the European Community by directive 91/1843/EEC, and similar concerns have been cited by the EPA. Concern regarding the high incidence of food adulteration with Rhodamine B outside of the EC has also been reported [12].

Rhodamine B is also widely used as a tracer in water analysis [2,13,14] to determine the rate and direction of flow and transport. Questions on the possible adverse environmental effects of its use have therefore arisen [13,15]. These environmental water tracing studies are generally undertaken by absorbance [16] or fluorescent spectroscopy [17]. However, at low concentrations the determination of Rhodamine B can be hindered by naturally occurring florescent compounds which can limit the usefulness of this approach [2,18]. Its common use as an industrial dye has also led to environmental water contamination. Consequently, there is a pressing need for methods capable of detecting low concentrations of Rhodamine B in such samples.

In its initial conception, electroanalysis was founded on laboratory-based Hg working electrodes [19], leading to a lack of portability and issues with toxicity. A variety of other electrode materials have been consequently considered as possible alternatives, such as glassy carbon [20] and carbon paste [21] which necessitate skill in their preparation. More recently, the application of techniques such as screen printing has allowed for the highly accurate and precise mass production of carbon electrodes. This is exemplified by the widely used commercial personal glucose biosensor [22], the market for which has prompted numerous other screen-printed devices for an equally large number of applications [23,24,25,26,27]. The possibility of mass production allows for such devices to be treated as disposable, and when coupled with modern battery-powered potentiostats, screen-printed carbon electrodes (SPCEs) can be used in the field, at the point-of-need.

Rhodamine B has been studied at a number of different electrode materials [28,29,30,31,32,33,34,35]. However, it is believed that this study represents the first report on the voltammetric behaviour and determination of Rhodamine B in an environmental water sample at an unmodified SPCE. Numerous investigations have shown the possibility of modifying SPCEs by now well-established techniques [36]. However, such further fabrication steps can be argued to decrease the cheap mass production advantages that screen-printing can afford. It is also important to be able to understand the behaviour of an analyte at the working electrode prior to modification, as results can be misleading if there is not a good understanding of the base working electrode [37]. In previous investigations, it has been shown that SPCEs can, without further modification, be used for the determination of a number of different analytes, including trace environmental pollutants such as Pb [38], for forensic applications, and for determinations of drugs such as diazepam [39] and clonazepam [40]. It has also been shown that dyes, such as 1-(2-pyridylazo)-2-naphthol, can absorb to the surface of these same unmodified SPCEs [41]. Consequently, it is important to investigate the voltammetric behaviour of Rhodamine B at these unmodified SPCEs.

In the first part of this investigation, the cyclic voltammetric behaviour of Rhodamine B was investigated, and the effects of pH and scan-rate explored. The voltammetric behaviour of Rhodamine B at an SPCE was first investigated using a saturated calomel reference electrode. There being no previous reports of its behaviour at unmodified SPCEs the use of a standard reference electrode, as part of a three-electrode system, allowed for the results to be more readily directly compared with literature findings. A screen-printed working and pseudo-reference electrode were then used to explore the possibility of applying such devices as sensitive, disposable sensors for at-the-point of-need investigations of Rhodamine B.

## 2. Materials and Methods

### 2.1. Chemicals and Reagents

Unless otherwise stated, chemicals and reagents were obtained from Fischer Scientific (Loughborough, UK). Stock solutions of orthophosphoric acid, trisodium orthophosphate, disodium-orthophosphate and sodium orthophosphate were made at a concentration of 0.2 M by dissolving the required mass in deionised water and titrated together to give the desired pH buffer solution. Stock solutions of Rhodamine B were prepared by dissolving the appropriate mass in deionised water to give a 10 mM solution. Deionised water was obtained from a Purite RO200−Stillplus HP System, (Purite Oxon., UK). Standards, for voltammetric investigations, were prepared by dilution of the stock solution with phosphate buffer to give a 0.1 M phosphate buffer solution containing 1.0 mM Rhodamine B. Environmental water samples were obtained from an urban drainage holding pond, at the University of the West of England (UWE), Bristol, UK (51°29′55.6″ N 2°32′40.5″ W).

### 2.2. Apparatus

Cyclic voltammetric investigations were undertaken using a Model 263 EG&G Princeton Applied Research potentiostat (Princeton, NJ, USA) with EG&G electrochemistry software for data acquisition and control. The glass-coated platinum wire auxiliary electrode and the saturated calomel reference electrode (SCE) were obtained from Russell, Fife, UK. An SPCE, was employed as the working electrode; printed from C10903D14 (Gwent Electronic Materials Ltd., Pontypool, UK). The electrode was cut from the Valox substrate, and the working electrode area (9 mm^2^) defined with a strip of tape (RS Components, Corby, Northamptonshire, UK). Differential pulse voltammetry was undertaken with a Pstat10 potentiostat interfaced to a PC for control and data acquisition via the General-Purpose Electrochemical System Software Package version 3.4 (Autolab, The Netherlands). In these studies, a dual screen-printed system was used, with the same working electrode, but in this case with an Ag/AgCl track printed around it (2 × 0.2 cm^2^), serving as a combined pseudo-reference and counter electrode, the configuration of which has previously been given [27,42].

### 2.3. Voltammetric Procedures

Initial cyclic voltammograms were recorded in 10 mL solutions of 0.1 M phosphate buffer and then in the same solution containing 1.0 mM Rhodamine B, using a starting potential of 0.0 V, with an initial switching potential of −1.5 V and a second switching potential of +1.5 V with a final potential of −0.5 V. Solutions were degassed by purging with nitrogen (BOC, Guildford, UK). Differential pulse voltammograms were obtained over the potential range −0.2 V (held for 15 s) to +1.0 V using a step height of 2.4 mV, step width of 0.2 s, pulse amplitude of 50 mV, and pulse width of 50 ms.

### 2.4. Sample Preparation

A 2.5 mL aliquot of sample water was diluted with 2.5 mL of pH 7.1, 0.2 M phosphate buffer. One hundred µL aliquots of this were then examined using the optimised differential pulse voltammetric conditions described above, in Section 2.3. Quantification was obtained by external calibration.

## 3. Results

### 3.1. Cyclic Voltammetric Investigations

**Figure 1:**
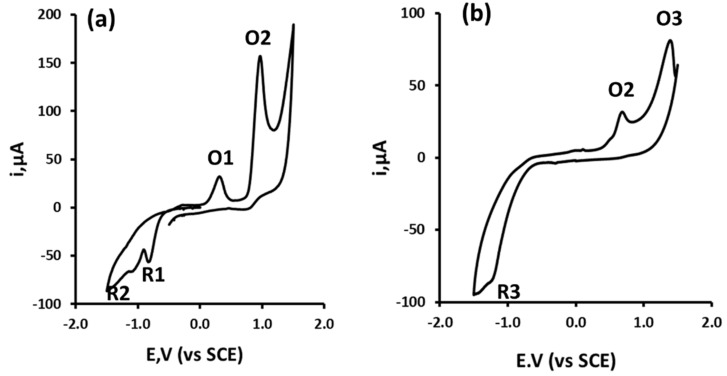
Cyclic voltammogram, obtained at a scan rate of 100 mVs^−1^, for a 1.0 mM solution of Rhodamine B in 0.1 M phosphate at pH 8.3. (**a**) Starting potential 0.0 V, initial switching potential −1.5 V, second switching potential +1.5 V, final potential −0.5 V and (**b**) second scan of the same SPCE. Voltammetric conditions as for Figure 1a.

Figure 1a shows the cyclic voltammogram of Rhodamine B obtained at pH 8.3. The voltammogram was obtained by first scanning negatively from 0.0 V to −1.5 V, then switching the scan direction and scanning to +1.5 V. The voltammogram was predominated on the negative-going scan by two reduction peaks designated as R1 and R2; and on the return positive scan by two oxidation signals, O1 and O2. If the scan was repeated using the same conditions as Figure 1, a new voltammetric wave, O3 is also recorded, as shown in the second repeated scan (Figure 1b). A different signal reduction process (R3) was also observable, with a peak potential (Ep) of −1.0 V. The process O2 was still recorded, but O1 no longer occurred. This new oxidation process, O3 was believed to result from the formation of a dimer [43] on the first voltammetric scan.

**Figure 2:**
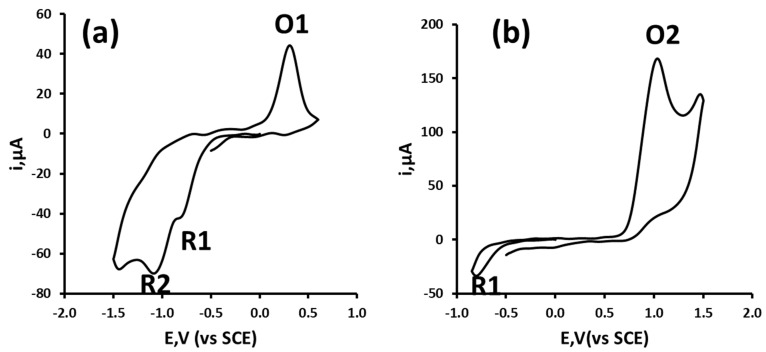
(**a**) Starting potential 0.0 V, initial switching potential −1.5 V, second switching potential +0.5 V, final potential −0.5 V and (**b**) starting potential 0.0 V, initial switching potential −0.7 V, second switching potential +1.5 V, final potential −0.5 V.

Further investigations of the behaviour of the O1 process were undertaken by changing the switching potential to 0.6 V, at a point before the occurrence of the oxidation peak, O2. Previous reports at a platinum foil electrode at low pH values [44,45] showed the presence of a quasi-reversible couple with Ep values of +1.2 V (vs. NHE) and +1.11 V (vs. NHE) formed following the voltammetric oxidation of Rhodamine B. However, as shown in this study (Figure 2a), by scanning in the negative direction following the oxidation process, O1, no new reduction peaks were recorded. The reduction process, R2, and the oxidation process, O1, are quasi-reversible, but exhibited a much greater degree of separation of 1.46 V than the redox couple recorded in the previous study [44,45] at the platinum foil electrode. These were consequently concluded to be the result of a different process from that reported previously [44,45].

It was thought that the oxidation process occurring at O1 could result from a species formed at either R1 or R2. To investigate this further, we studied the effects of changing the switching potential, in this case from −1.5 V to −0.7 V, i.e., before the start of the process occurring at R2. From the resulting voltammogram, shown in Figure 2b, it was evident that the O1 process could no longer be seen on the return positive-going scan when using this switching potential. It was concluded that this demonstrated the dependence of this O1 species on the reduction process occurring at R2. Extension of the scan to +2.5 V or −2.5 V did not show any evidence of any further redox processes.

### 3.2. Effect of pH on Peak Potential and Peak Current

**Figure 3:**
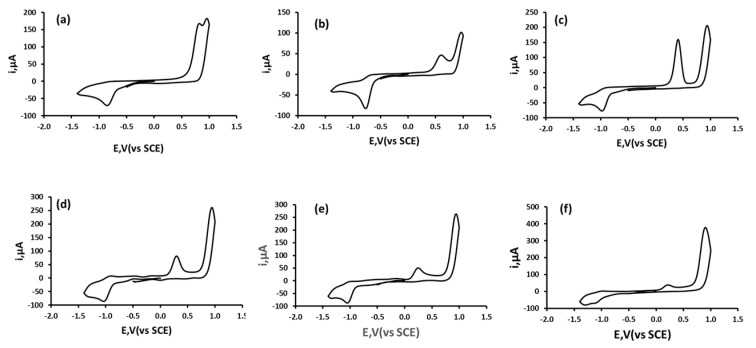
Typical cyclic voltammograms obtained for 1.0 mM Rhodamine B in (**a**) pH 1.92, (**b**) 3.51, (**c**) pH 7.17, (**d**) pH 8.00, (**e**) pH 8.78 and (**f**) pH 11.4.

**Figure 4:**
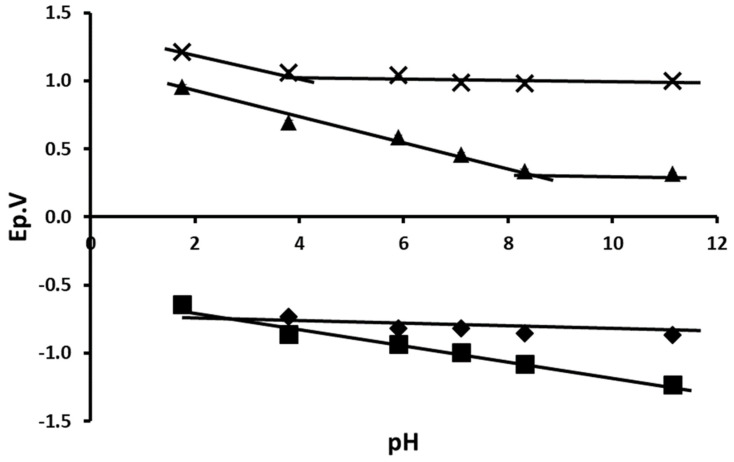
Plot of Ep vs. pH for Rhodamine B. Crosses O2; triangles O1; diamonds R1 and squares R2. Voltammetric conditions as Figure 1.

**Figure 5:**
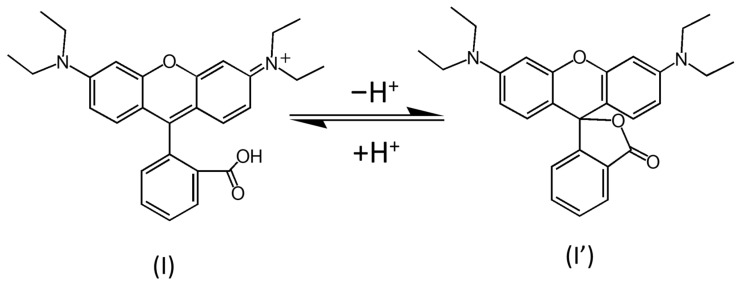
The pH dependence for the spirolactam form of Rhodamine B.

Figure 3 shows the typical cyclic voltammograms obtained over the pH range investigated. The voltammograms were predominated by two oxidation peaks, O1 and O2, and two reduction peaks, designated as R1 and R2. The relationship between Ep and pH over the range pH 2 to pH 12 is shown in the plots in Figure 4. The reduction process R2 followed a near theoretical relationship with pH to that predicted by the Nernst equation, giving a slope of 62 mV/pH. The oxidation signal, O1, gave a slope similarly close to the theoretical 59 mV/pH, at 68 mV/pH, indicating an even number of electrons and protons involved in the reduction and oxidation processes of these species. The oxidation peak, O2, was found to have a slope of 83 mV/pH between pH 2 and pH 4, and then became independent of pH above these values. This break-point in the plot is believed to reflect the reported pk_a_ value of Rhodamine B of 4.3 [46]. It is known that Rhodamine-type dyes exhibit a pH-dependent equilibrium between two forms, a coloured and fluorescent form in acidic conditions (I), and a colourless and nonfluorescent spirolactam form (I’) at higher pH values (Figure 5). As it has been shown that this pH-related change in structure has marked effects on both the fluorescence and the colour of the compound [47]. It is believed that this also results in changes in the cyclic voltammetric behaviour of Rhodamine B recorded for the oxidation of tertiary amine (O2).

**Figure 6:**
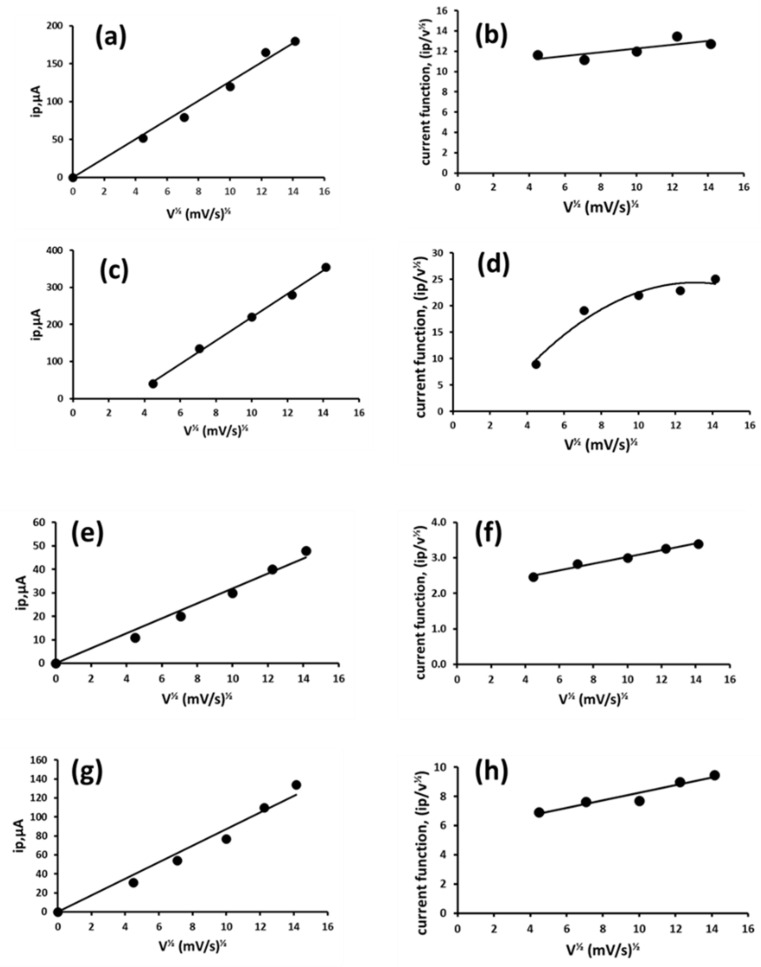
Plots of peak current vs. square root of scan rate for (**a**) O1, (**c**) O2, (**e**) R1 and (**g**) R2. Plots of current function for (**b**) O1, (**d**) O2, (**f**) R1 and (**h**) R2. Other voltammetric conditions as for Figure 1a.

The reduction process, R1, was found to be independent of pH across the range studied. The effect of scan rate over the range 20 mVs^−1^ to 200 mVs^−1^ was studied at pH 8.3. Apart from the oxidation peak, O2, all of the principle peaks investigated were found to be linearly dependant on the square root of scan rate (*v*^½^), demonstrating the voltammetric processes to be diffusion-controlled (Figure 6). Further examination of current function (ip/*v*^½^) showed that peaks O1, R1 and R2 exhibited diffusion-controlled behaviour. Examination of the current function plots for the O2 process showed a possible electrochemical chemical oxidation type behaviour [48] (Figure 6d).

**Figure 7:**
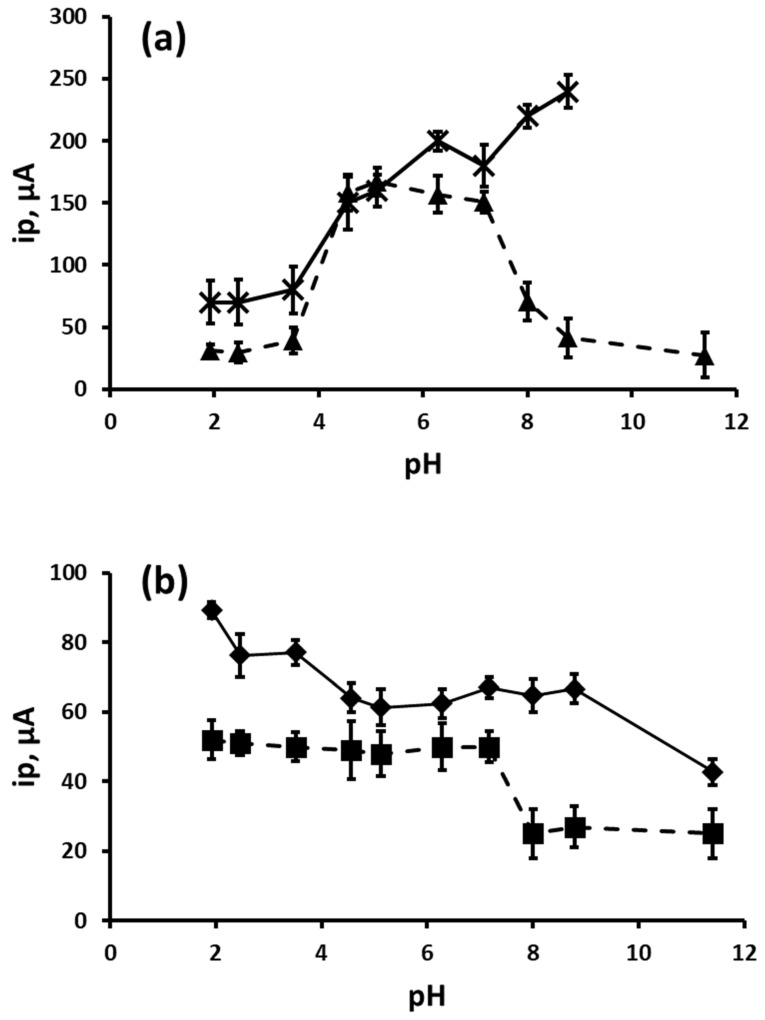
Plot of *i*_p_ vs. pH for Rhodamine B (**a**) oxidation processes, O1 and O2 and (**b**) reduction processes, R1 and R2. Crosses O2; triangles O1; diamonds R1 and squares R2. Voltammetric conditions as Figure 1. Error bars represent plus or minus a standard deviation.

Plots of *i*_p_ vs. pH for the principle peaks recorded by cyclic voltammetry (Figure 7) shows that the ip for all the peaks investigated varied with changing pH across the range investigated. Interestingly, the oxidation peak, O1, showed a similar profile to that seen for the related dye malachite green [49], showing a maximum between pH 4 and 7. The change in behaviour at pH 4 is believed to reflect the pKa of Rhodamine B. The oxidation peak, O2, was seen to increase in magnitude as the pH of the supporting electrolyte was increased. This peak is believed to result from the direct oxidation of Rhodamine B. It is believed that the change in the voltammetric response is due to the changing interaction of the acid group (Figure 5) with the rest of the molecule as the pH is increased.

**Figure 8:**
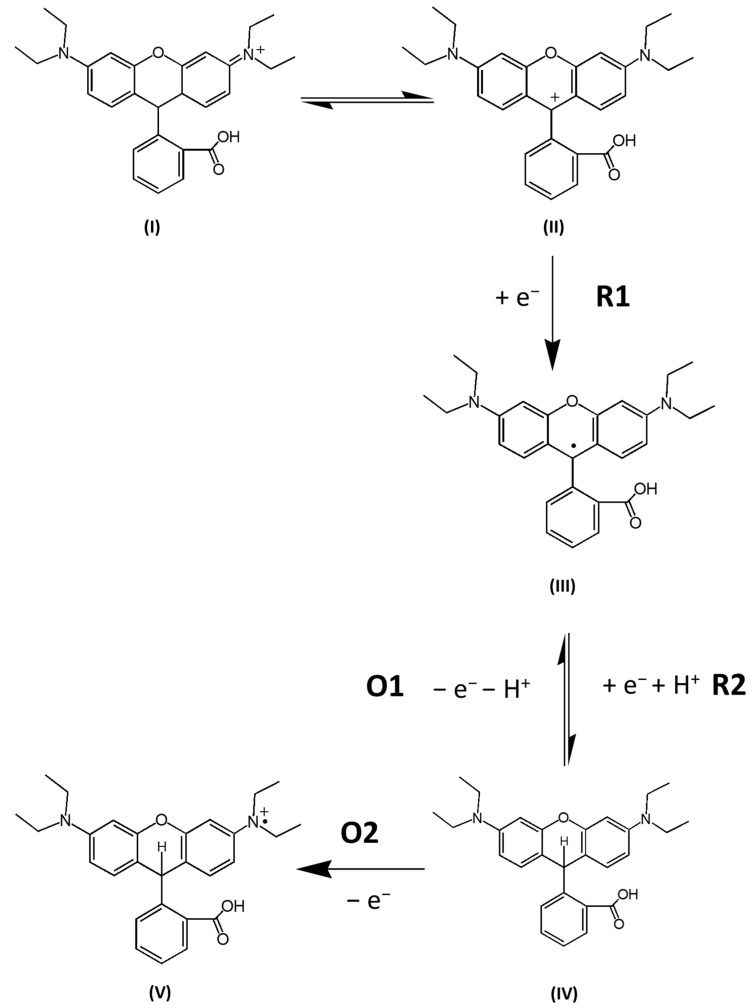
Proposed scheme for the voltammetric behaviour of Rhodamine B at the SPCE.

Figure 8 shows the proposed electrochemical reaction scheme to explain the main peaks observed for the cyclic voltammetric behaviour of Rhodamine B. If the cyclic voltammogram obtain at pH 8.3 in Figure 1a is considered, it is believed that R1 is the result of a one-electron reduction of the resonance species ((I, II) to from the radical species (III). R2 is the result of the reduction of the radical species (III) through a one-electron, one-proton reduction to give species (IV). This is in a similar manner to that described by Compton et al. [50] for the electrochemical reduction of the closely related dye, fluorescein. In their study the two reduction processes were believed to be in some form of equilibrium. However, Okada et al. [51], in their voltammetric study of Rhodamine B, showed the same voltammetric reduction process as irreversible. In the present study, the subsequent quasi-reversible oxidation of species (IV) on the return positive-going scan then occurred at O1, through an overall one-electron, one-proton oxidation back to (III). The oxidation process, O2, occurred through a separate one-electron oxidation of the amine to give (V).

### 3.3. Calibration Plot, Limit of Detection and Precision

A calibration study was carried out for Rhodamine B, over the range 0.50–1025 µgmL^−1^, using the cyclic voltammetric conditions described above, but in this case at pH 7.1, approximate to that expected for environmental and potable water. For the process, O1, the plot was linear over the range studied with a slope of 0.0899 µA/µg^−1^ mL^−1^, and an R^2^ value of 0.9929. Replicate determinations of an 8.2 µgmL^−1^ Rhodamine B solution using three separate SPCEs gave a coefficient of variation of 5.6%. In the second oxidation process, O2 demonstrated a quasi-linear relationship over the concentration range investigated. A polynomial relationship (y = −0.0038x^2^ + 0.81x) was obtained over the concentration range 1.0 µgmL^−1^ to 102 µgmL^−1^, with an associated R^2^ value of 0.9914. The coefficient of variation for three separate SPCEs for this signal was found to be 3.5% for a Rhodamine B concentration of 8.2 µgmL^−1^. The limit of detection using this second oxidation peak was calculated to be 0.4 µgmL^−1^.

**Figure 9:**
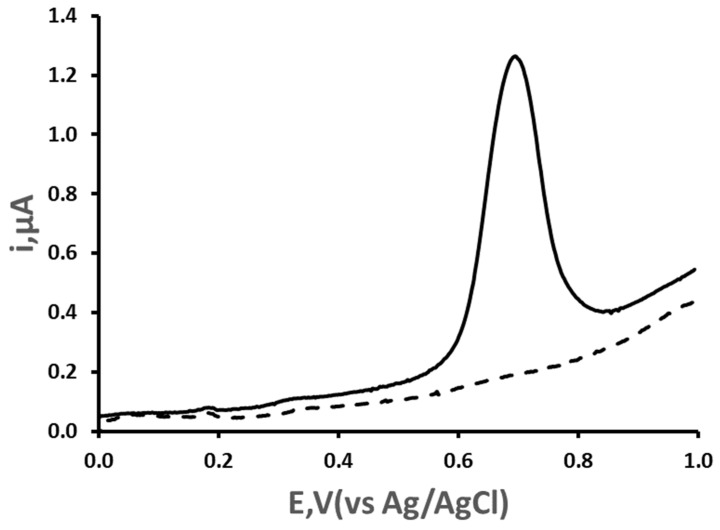
Differential pulse voltammograms obtained with an SPCE for solid line, 2.1 µgmL^−1^ Rhodamine B in 0.1 M phosphate buffer pH 7.1. Dashed line, buffer only. Voltammetric conditions: 0.0 V 15 s vs. Ag/AgCl. Pulse repetition time 0.2 s, step height 2.4 mV in the positive potential by applying pulse amplitude of 50 mV, pulse duration 50 ms.

**Figure 10:**
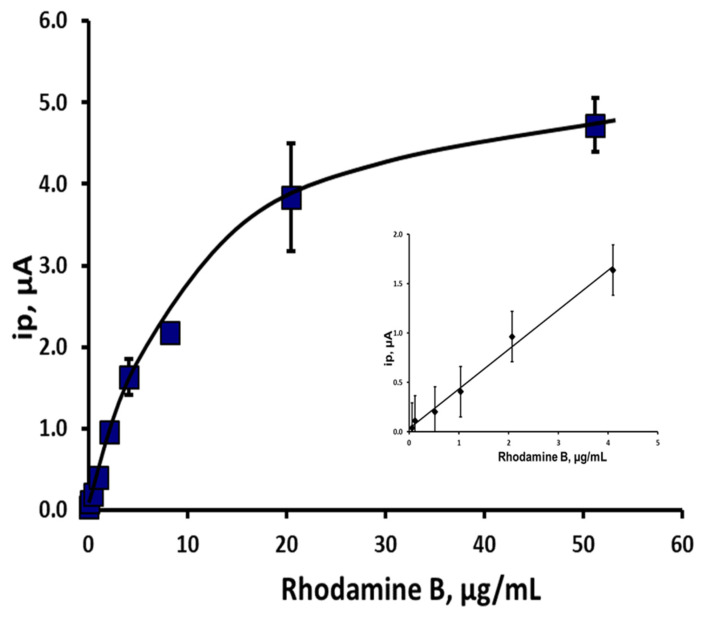
Effect of Rhodamine B concentration on the differential pulse voltammetric current peak (*i*_p_) for oxidation peak O2. Insert, linear section. Each point is the mean of three separate SPCEs. Error bars represent plus and minus a standard deviation.

One of the main limitations in terms of sensitivity, of cyclic or linear sweep voltammetry, lies in the significant contribution of capacitance current to the overall current recorded. Differential pulse voltammetry offers a better differentiation of the analytical, faradic current against the background capacitance current generated as the potential of the working electrode is changed. In order to ascertain whether the detection limit could be improved, an examination of this more sensitive waveform was undertaken. Differential pulse voltammetric studies were undertaken over the concentration range 0.06 µgmL^−1^ to 500 µgmL^−1^. Figure 9 shows a typical differential pulse voltammogram of a 2.1 ppm Rhodamine B standard obtained under the optimised conditions. This shows a well-defined peak with an Ep of +0.71 V corresponding to the O2 process seen by cyclic voltammetry in Figure 1. The response was linear between 0.06 and 4.0 µgmL^−1^ with a slope of 0.420 µA µg^−1^ mL^−1^ R^2^ = 0.9783 (Figure 10). Coefficients of variation of 3.7% and 2.6% were obtained for Rhodamine B concentrations of 0.06 µgmL^−1^ and 8.2 µgmL^−1^, respectively, using this approach.

### 3.4. Effect of Ascorbic Acid on Rhodamine B Differential Pulse Voltammetric Response

A number of reports have highlighted the electrochemical interactions that dyes, such as Rhodamine B exhibit with ascorbic acid [4,52,53,54,55,56]. Studies have shown that Rhodamine B [57,58,59,60,61], and a number of other related dyes [62,63,64], can form reactive oxygen compounds following the dyes’ transformation to their excited state by the absorption of light [65]. In a recent study, Stanley et al. [66] investigated the possibility of using ascorbic acid as a scavenger for the O_2_^−^ and ^•^OH species formed in this process. Their investigation showed that ascorbic acid reduced dye degradation by 75%. Hence, it is believed that in the presence of light, Rhodamine B undergoes this photochemical reaction and the corresponding formation of reactive oxygen species. This can result in the oxidisation of Rhodamine B via its amine groups [60]. These reactions, hence, lead to a depletion of the Rhodamine B available for electrochemical oxidation and a smaller analytical signal. The addition of an antioxidant such as ascorbic acid can, alleviate these photochemical and chemical oxidations. This allows for a greater proportion available for electrochemical oxidation, enhancing the voltammetric oxidation peak designated as O2.

Investigations were undertaken into the effect of Rhodamine B concentration on a fixed 1.0 mM concentration of ascorbic acid. An enhancement of the differential pulse response of ascorbic acid was recorded, which followed a linear relationship (slope = 0.0019 µA ng^−1^ mL^−1^, R^2^ = 0.9858) over the Rhodamine B concentration range 0.24 µgmL^−1^ to 1.0 µgmL^−1^.

**Figure 11:**
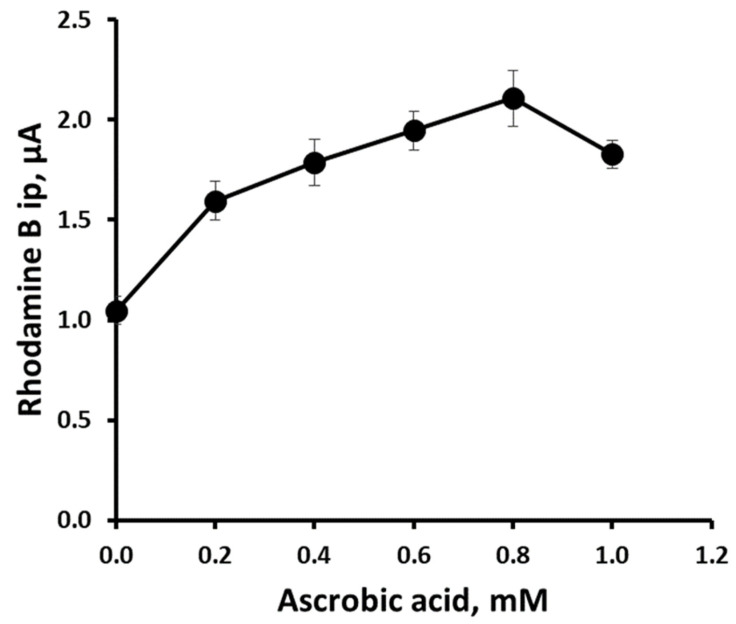
Differential pulse voltammograms peak currents obtained with an SPCE for 2.1 µgmL^−1^ Rhodamine B in 0.1 M phosphate buffer pH 7.1 with increasing concentrations of ascorbic acid. Voltammetric conditions as for Figure 7. Each point is the mean of three individual SPCEs. Error bars represent plus and minus a standard deviation.

**Figure 12:**
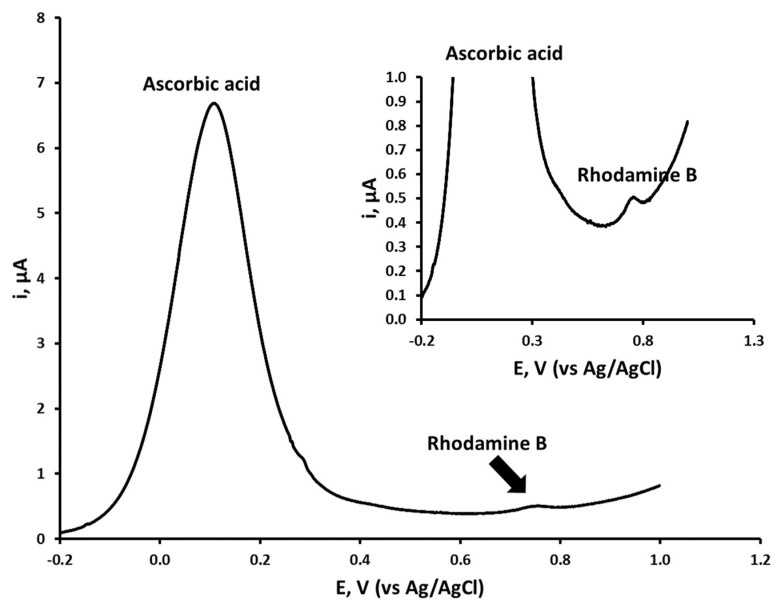
Differential pulse voltammogram of Rhodamine B in an environmental water sample adjusted to be 0.1 M pH 7.1 phosphate buffer, 0.8 M ascorbic acid. Insert shows same voltammogram at larger scale.

Further studies were undertaken to investigate the effect of varying the concentration of ascorbic acid on the differential voltammetric response of a fixed 2.0 µgmL^−1^ concentration of Rhodamine B in a 0.1 M pH 7.1 phosphate buffer (Figure 11). The *i*_p_ of Rhodamine B was found to increase with ascorbic acid concentration up to 0.8 mM, beyond which the Rhodamine B response began to decrease. Consequently, further differential pulse voltammetric investigations were undertaken using a fixed ascorbic acid concentration of 0.8 mM. Figure 12 shows a representative differential pulse voltammogram of an environmental water sample fortified to be 96 µg/L Rhodamine B, adjusted to be 0.1 M pH 7.1 phosphate buffer, 0.8 M ascorbic acid.

Table 1 provides a comparison of the optimised DPV method with previously reported analytical methods for the determination of Rhodamine B. The linear range for the method is comparable to that reported by high performance liquid chromatography following solid phase extraction [17] and that recorded for modified SPCEs [29]. The quoted limit of detections for the spectroscopy-based approaches [67,70,73] are lower than those for the voltammetric method developed here, however, these necessitate time-consuming, complex sample pre-treatment steps. The differential pulse voltammetric method described here requires little sample pre-treatment, other than dilution with phosphate buffer and ascorbic acid. The developed method is hence simple and fast. The application of a disposable SPCE also allows for the method to be used at the point-of-need or in the field.

### 3.5. Analytical Application

The optimised procedure was used to determine the concentration of Rhodamine B in a spiked and unspiked environmental water sample. A fixed ascorbic acid concentration of 0.8 mM was used throughout. The recovery and precision data obtained by this approach is summarised in Table 2. Clearly, these results show that this approach of monitoring the enhancement of the differential pulse response of Rhodamine B by ascorbic acid at the SPCEs offers the possibility of determining such dyes with little sample preparation.

## 4. Conclusions

A simple and convenient differential pulse voltammetric assay for Rhodamine B was developed, based on an SPCE via the interaction of Rhodamine B with ascorbic acid using sample volumes of only 100 µL. A detection limit of 10 ngml^−1^, with a linear response up to 1.0 µgmL^−1^ (R^2^ = 0.9858) was achieved using an ascorbic acid concentration of 0.8 mM. The results shown in Table 2 indicate that the method gives reliable results using an external calibration method. The analytical performance characteristics were better than those reported at a glassy carbon electrode [28] and an Ag nanoparticle modified screen-printed electrode [29]. It was also shown possible to determine Rhodamine B via cyclic voltammetry using the same SPCE over the range 0.50–1025 µgmL^−1^ (R^2^ = 0.9929).

As far as is known, this is one of the few reports to explore more deeply the voltammetric behaviour of Rhodamine B, exploring the behaviour of processes beyond its direct electrochemical oxidation. Rhodamine B was found to exhibit two oxidation peaks and two reduction peaks, designated O1 and O2, and R1 and R2, respectively. The oxidation process, O1, was found to be dependent on the previous reduction of Rhodamine B via a two-step reduction process (R1 and R2) to the leuco-rhodamine derivative. The O2 oxidation process was concluded to result from the direct oxidation of the tertiary amine group and was found to be independent of R1 and R2.

This is also the first report on the use of differential pulse voltammetry employing an unmodified SPCE for the trace determination of Rhodamine B in an environmental water sample. Future studies will focus on extending the application of the optimised method. Preliminary studies have shown the possibility of using the same approach for the determination of Rhodamine B in alcoholic beverages and chili powder.

## Figures and Tables

**Table 1:** Performance comparisons of the detection of Rhodamine B with other methods.

Sample	Linear Range µg/L	Limit of Detection µg/L	Technique	Comments	Reference
Wastewater and surface water	50–1000	0.5	High-performance liquid chromatography with fluorescence detection	Solid-phase extraction	[17]
Fruit juice and Preserved fruit	4.78–956.1	2.93	Differential pulse voltammetry at a glassy carbon electrode	Preserved fruits extracted in water with the aid of ultrasonication for 4 h, and then filtrated under vacuum	[28]
Chili powder and tomato sauce	5–100	1.44	Magnetic solid-phase extraction, with SPCE modified by multiwalled carbon nanotubes and a molecular imprinted polymer	Chili powder extracted with acetonitrile with the aid of ultrasonication. Centrifuged and mixed with NaCl and distilled water, stored at −20 °C for 2 h. Filtrated, under nitrogen, and reconstituted with distilled water at pH 5. Tomato sauce extract obtained via direct filtration	[30]
Tomato and chili sauces	0.96–44.07	1.79	Zeolite imidazolate framework-67/reduced graphene oxide modified glassy carbon electrode	Sample was sonicated in water by ultrasonication and stirring The Rhodamine B concentration was then determined using the optimized conditions	[33]
Water samples and hair colors	48–720	9.6	Multi-walled carbon nanotube carbon paste electrode	Hair color was sonicated in ethanol and diluted with PBS (pH = 3.0) and examined electrochemically	[34]
Water samples and soft drinks	23–2000	7.0	Spectrophotometric detection	Dispersive liquid–liquid microextraction	[67]
Chili powder	1.0–10,000 µg/g	1.0 µg/g	Surface-enhanced Raman spectroscopy	Extraction with acetonitrile via shaking, sonication and centrifuging	[68]
Bakery products, beverages and cooked foods	600–5000	10	High-performance liquid chromatography	Officially prescribed method of the Indian food regulatory authority	[69]
Cosmetics	0.765–478.03	0.239	Micellar-enhanced fluorimetry	Lipstick extracted in water by stirring at 333 K (ca. 60 °C) for 15 min	[70]
Surface water	--	0.000010	High-performance liquid chromatography with fluorescence detection	Solid-phase extraction of 1 L of sample water reconstituted in a 1.0 mL of mobile phase	[71]
Soft drink, waste water and cosmetics	250–3000	3.14	Spectrophotometry at 556 nm	Solid-phase extraction	[72]
Cosmetic products and water samples	--	2.2	Spectrophotometry at 550 nm	Deep eutectic solvent-based liquid-phase microextraction	[73]
Chili powder, tomato juice, soy sauce and pasta sauce	144–1440	48	Cu@CS nanohybrid-modified glassy carbon electrode	Tomato sauce extract was obtained by filtration. Chili powder was sonicated in acetonitrile and centrifuged. NaCl and water were added and the mixture frozen and centrifuged. The acetonitrile layer collected. Soy sauce was sonicated in a mixture of ethanol/water/acetic acid, and filtered. Pasta sauce was diluted in water and filtered. Once processed, extracts were made up in pH 6.5 PBS, and examined using the optimized procedure	[74]
Chili powder and preserved fruit	5–2400	2.1	Silica-pillared zirconium phosphate/Nafion composite modified glassy carbon electrode	Sample added to acetone–n-hexane solution and sonicated. Mixture then centrifuged and supernatant extracted with n-hexane and organic phase discarded following addition of water. An aliquot was then diluted in pH 5.0 BR buffer and investigated using the optimized conditions	[75]
Surface water	60–4000	10	Differential pulse voltammetry at an unmodified SPCE	Dilution with phosphate buffer and addition of ascorbic acid	This work

**Table 2:** Recovery and precision data obtained for Rhodamine B. Mean recovery of 94.3% with an associated coefficient of variation of 2.9%. ND = not detected.

	Original Concentration ng/mL	Added ng/mL	Found ng/mL	% Recovery
1	ND	96.0	90.0	93.8
2	ND	96.0	90.5	94.3
3	ND	96.0	89.9	93.6
4	ND	96.0	87.1	90.7
5	ND	96.0	95.3	99.3

## Data Availability

Not applicable.
